# Epidemiology and molecular characterization of carbapenem-resistant *Klebsiella pneumoniae* isolated from neonatal intensive care units in General Hospital of Ningxia Medical University, China, 2017–2021

**DOI:** 10.1007/s10123-024-00510-0

**Published:** 2024-03-21

**Authors:** Zhuoran Qiu, Yuting Kang, Chao Xu, Wanting Ma, Gang Li, Wei Jia, Pengtao Wang

**Affiliations:** 1 College of Clinical Medicine, Ningxia Medical University, Yinchuan, 750004 Ningxia China; 2 Ningxia Key Laboratory of Clinical and Pathogenic Microbiology, Institute of Medical Sciences, General Hospital of Ningxia Medical University, Technology Building 602, 804 Shengli Road, Yinchuan, 750004 Ningxia China; 3 Center of Medical Laboratory, The General Hospital of Ningxia Medical University, Technology Building 601, 804 Shengli Road, Yinchuan, 750004 Ningxia China

**Keywords:** CRKP, Colonization, Infectious disease, Antibiotic-resistance, Virulence plasmid

## Abstract

**Objectives:**

This study aimed to retrospectively investigate the epidemiology and molecular characteristics of carbapenem-resistant *Klebsiella pneumoniae* (CRKP) isolates from neonatal intensive care units (NICU) between 2017 and 2021.

**Methods:**

The antibacterial susceptibility of all strains was assessed using the VITEK 2 compact system. The presence of antibiotic resistance, virulence genes, sequence types (STs), capsular (K) types, and the *wzi* genes was determined through polymerase chain reaction (PCR). Molecular typing was performed by pulsed-field gel electrophoresis (PFGE) using the restriction enzyme XbaI. Additionally, the virulence potential of *peg344*-positive strains was evaluated using the string test and mouse intraperitoneal infection models. Whole-genome sequencing was conducted on the DNB system and PacBio platforms.

**Results:**

A total of 46 CRKP isolates were collected during the study period. Out of these, 93.47% (43/46) were identified as CRKP strains belonging to the ST76-K10 type carrying *bla*_NDM-5_. It was observed that CRKP infection resulted in more severe clinical symptoms compared to CRKP colonization. Among the CRKP strains, a hypervirulent CRKP strain called KP-63, belonging to the ST23 type, was identified. This strain exhibited high mortality in the mouse infection model and was found to possess virulence genes. Genomic alignment analysis revealed a significant similarity between the virulence plasmid from KP-63 strain (pKP-63) and pK2044 from the hypervirulent *K. pneumoniae* strain NTUH-2044.

**Conclusions:**

There has been a potential dissemination of ST76-K10 type CRKP carrying *bla*_NDM-5_ in the NICU at Ningxia Hospital. Neonatal CRKP infection has been found to cause more severe clinical symptoms than colonization. Furthermore, we have discovered a CR-hvKP strain of ST23 with serotype K1, which exhibits a significant resemblance in its virulent plasmid to pK2044. Therefore, it is crucial to enforce effective measures to restrict the spread and hinder the evolution of CRKP within the hospital.

## Introduction

Due to the underdeveloped neonatal immune system, limited colonization of protective flora, and influence of maternal factors, newborns are vulnerable to serious and life-threatening infections caused by various types of bacteria (Sampah and Hackam 2020). This vulnerability is particularly evident in low-birth weight neonates admitted to the neonatal intensive care unit (NICU). The use of invasive procedures and frequent exposure to multiple antibiotics can contribute to the emergence and spread of drug-resistant bacteria among them (Milton et al. 2022), leading to increased morbidity and mortality, longer hospital stay, and a higher economic burden. Therefore, it is crucial to investigate the molecular epidemiology and characterization of these drug-resistant bacteria isolated in the NICU population. This research aims to provide comprehensive and reliable information for the prevention and treatment of neonatal infectious diseases.

The prevalence of carbapenem-resistant *Enterobacteriaceae* (CRE) infections, particularly carbapenem-resistant *Klebsiella pneumoniae* (CRKP), is increasing globally, leading to high levels of morbidity and mortality (van Duin et al. 2020). CRKP refers to *K. pneumoniae* strains that are resistant to carbapenems. Since its first isolation in Germany in 1985 (Knothe et al. 1987), CRKP has been progressively spread globally, posing a substantial threat to public health (Yang et al. 2021). A recent meta-analysis revealed that the global prevalence of CRKP colonization varies from 0.13% to 22%, with an overall pooled prevalence of 5.43%. Likewise, the incidence of CRKP colonization ranges from 2% to 73%, with a pooled incidence of 22.3% (Tesfa et al. 2022). The mortality rate associated with CRKP infections is estimated to be between 33% and 42% (Agyeman et al. 2020; Xu et al. 2017). The most prevalent sequence type of clinical *K. pneumoniae* globally is ST258, primarily due to its rapid dissemination and multidrug resistance properties. However, in China, the dominant clone is ST11, and the majority of these isolates produce *K. pneumoniae* carbapenemase (KPC) (Q. Wang et al. 2018).

A hypervirulent clinical variant of *K. pneumoniae*, known as hvKP, has been identified in Taiwan since the early 1990s. HvKP has the ability to cause severe invasive infections, such as liver abscesses, meningitis, endophthalmitis, and necrotizing fasciitis, even in healthy individuals. It is important to note that hvKP is vulnerable to antimicrobials (Prokesch et al. 2016). A study conducted in China found that the vast majority (90.9%) of the pathogens responsible for pyogenic liver abscesses were hypervirulent *K. pneumoniae* (Ye et al. 2016). Moreover, the clinical importance of this pathogen has also been emphasized in recent research conducted in the USA and European nations (Kocsis 2023). HvKP strains are commonly linked to sequence types (STs) 23, ST65, and ST86, with the main serotypes of the capsules being K1 and K2 (Lan et al. 2021). However, gene transfer between CRKP and hvKP isolates results in the emergence of carbapenem-resistant hypervirulent *Klebsiella pneumoniae* (CR-hvKP) isolates which have been increasingly reported in recent years (Shao et al. 2021; J. Turton et al. 2019; J. F. Turton et al. 2018). Outbreaks of CR-hvKP have been documented in Chinese hospitals in 2014 and 2016, leading to significant clinical consequences (Gu et al. 2018). Additionally, hospital-based infections and transmission of CR-hvKP have been reported in other countries, including India, Iran, Singapore, and the USA (Cejas et al. 2014; Mohammad Ali Tabrizi et al. 2018; Octavia et al. 2019; Remya et al. 2018). The continuous evolution of plasmids encoding carbapenem resistance or hypervirulence led to the coexistence of carbapenem-resistant and hypervirulent traits in the same *K. pneumoniae* strain. This strain can cause community-acquired infection in healthy individuals and is difficult to treat with current antibiotics.

In this study, we conducted an investigation into the distribution of CRKP isolates from the NICU of the General Hospital of Ningxia Medical University between 2017 and 2021. The main objective was to examine the molecular and epidemiological characteristics of these isolates, with a particular focus on their antibiotic resistance and virulence traits.

## Methods and materials

### Patient classification and data collection

This study enrolled patients admitted to the NICU at the General Hospital of Ningxia Medical University in Ningxia, China, between 2017 and 2021. Data was collected on various factors including sex, age, gestational age, body weight, height, body mass index, underlying conditions, antimicrobial treatment, source of positive culture, outcome, days of hospitalization, NICU hospitalization, and hospitalization prior to CRKP isolation. CRKP infection was defined according to the standards developed by the Centers for Disease Control and Prevention, which manifested in patients through clinical signs and symptoms of infection. Patients who had more than one culture sample growing CRKP but did not develop an infection during hospitalization, and where CRKP was isolated after significant improvement in the patient’s condition, were classified as part of the colonization group. All the classification was confirmed by infectious disease specialists.

### Collection and drug susceptibility test of CRKP strains

A total of 46 non-repetitive CRKP strains were collected from the General Hospital of Ningxia Medical University’s NICU between 2017 and 2021. The strains were identified and subjected to antimicrobial susceptibility tests using the VITEK2 Compact automatic microbial analyzer (BioMerieux, Paris, France), following the guidelines outlined in document M100-S26 established by the Clinical and Laboratory Standards Institute. Prior to participation, informed consent was obtained from each patient or their proxy in cases where the patient was incapable of providing consent.

### Detection of antibiotic-resistance genes

All isolates were screened by PCR for carbapenemase and other β-lactamase genes, including *bla*_KPC_, *bla*_NDM_, *bla*_IMP_, *bla*_VIM_, *bla*_TEM_, *bla*_SHV_, and *bla*_OXA-48_, as previously described (Eckert et al. 2006; Edelstein et al. 2003; Fursova et al. 2021). The PCR products were identified by agarose gel electrophoresis. *K. pneumoniae* isolates containing *bla*_KPC_ or *bla*_NDM_ were sequened for subtyping.

### String test

CRKP strains were incubated overnight on blood agar. A single colony was touched with a loop and stretched outward, and the length of the resulting viscous string was measured. A positive string test result was indicated by the formation of viscous strings longer than 5 mm.

Detection of virulence genes and capsular type identification

PCR was utilized to detect 10 genes associated with *K. pneumoniae* virulence, namely *rmpA*, *rmpA2*, *peg344*, *iucA*, *iroB*, *fimH*, *mrkD*, *iroN*, *entB*, and *magA* (Fursova et al. 2021; Bulger et al. 2017; Russo et al. 2014). The *wzi* genes were sequenced, and K types were determined following the method described by Pierre and Marie Curie University (Brisse et al. 2013) and Health Protection Agency in London (J. F. Turton et al. 2010). The *K. pneumoniae* sequence typing database (http://bigsdb.web.pasteur.fr) was utilized to identify the *wzi* alleles and K types.

### Pulsed-field gel electrophoresis and multilocus sequence typing

Pulsed-field gel electrophoresis (PFGE) was used to analyze the affinities between isolates of various species specimens. Bacterial DNA was treated with proteinase K and then lysed with the restriction enzyme XbaI at a temperature of 37°C for 2.5 h. This process resulted in a smaller quantity of larger DNA fragments. The XbaI-digested DNA was subjected to electrophoresis for 18.5 h at a voltage of 6 V, with a pulse angle of 120° and pulse times ranging from 6.8 to 35.4 s. The resulting patterns were analyzed and interpreted using the dice coefficient. In brief, strains were classified as belonging to clustered subtypes if they exhibited one to three fragment differences with a similarity of over 80%. Identical DNA fragmentation patterns were considered to be in the same classification. Multilocus sequence typing (MLST) analysis was performed on 46 CRKP strains using PCR methods to detect seven housekeeping genes (*rpoB*, *gapA*, *mdh*, *pgi*, *phoE*, *infB*, and *tonB*), following the protocol outlined in the Pasteur Institute and University College Cork’s provided primers and protocols (http://bigsdb.pasteur.fr/klebsiella/primers_used.html) (Cheng et al. 2018; Diancourt et al. 2005). The obtained products were sequenced at Tsingke Biotechnology in Beijing, China. Upon uploading and comparing the sequencing results, the allele number and sequence type (ST) were determined. The BioNumerics program was used to study population diversity and the relationship between MLST sequence types (STs).

### Mouse intraperitoneal infection models

This study was conducted in strict accordance with the recommendations in the Guide for the Care and Use of Laboratory Animals of the National Institutes of Health, and approved by the Ethics Committee for the Use of Laboratory Animals of Ningxia Medical University (Z2019/023, approved on 10 November 2019). A total of 40 female ICR mice, aged 6 to 7 weeks, were obtained from Huachuang Sino Company, and randomly divided into four groups. The bacteria were grown in LB broth until the logarithmic phase, and each mouse was injected with 5×10^7^ CFU bacteria. The negative control group received PBS, while the positive control group received the NTUH-2044 isolate. Mortality rates were observed for 48 h.

### Whole-genome sequencing

Genomic DNA was extracted from the KP-63 strain and subjected to whole genome sequencing using the DNB system, which generates 350-bp paired-end sequences, and the PacBio System, which assembles a 10-kb fragment library. To ensure more accurate and reliable results when sequencing on the Pacbio platform, the reads were filtered to remove low-quality data and adapter sequences. Before assembling, K-mer analysis was performed to estimate the genome size, degree of heterozygosity, and degree of duplication. De novo genome assembly was accomplished using SPAdes Genome Assembler (version 3.11.0). The predicted genes were annotated using the RAST tool (version 2.0) and Prokka (version 1.12.21). Plasmid maps were generated using GenomeVx (http://wolfe.ucd.ie/GenomeVx/). The circular plasmid map comparison was performed using the BLAST Ring Image Generator (version 0.95), while linear alignments of multiple genomic loci were conducted using EasyFigure (version 2.2.3). Plasmid incompatibility typing was identified by VRprofile 2.0 (https://tool2-mml.sjtu.edu.cn/VRprofile/).

### Statistical analysis

Statistical analysis was performed using the Staistical Package for the Social Sciences (SPSS) software, version 16.0. The data were presented as medians or means ± standard deviation. Statistical tests were two-sided and *P* < 0.05 was considered statistically significant.

## Results

### Clinical data of the patients and CRKP isolates

During the study period, a total of 46 patients were recruited, including 21 patients with CRKP infection and 25 patients with CRKP colonization. The mean gestational age of these patients was 33.54±3.98 weeks, and 24 (52.17%) patients were female. Most patients presented with severe underlying diseases, such as lung infection (95.65%), respiratory failure (63.04%), and newborn respiratory distress syndrome (32.61%). Notably, 92.86% of patients with sepsis had CRKP infection rather than CRKP colonization. There was a significant difference in the treatment with imipenem and amikacin sulfate between the CRKP infection and colonization groups (*P*<0.05). Patients with CRKP infection commonly received three or more antimicrobials during hospitalization. Our data showed a significant difference in NICU hospitalization between the two groups (*P*<0.05), with the CRKP infection group having a longer NICU hospitalization. Among the 46 CRKP isolates, they were primarily obtained from sputum (63.04%), catheter (13.04%), and blood (13.04%). The remaining isolates were obtained from urine (6.52%), secretion (2.17%), and shunt fluid (2.17%). Please refer to Table 1 for detailed clinical characteristics.

**Table 1:** Clinical characteristics of the patients in the neonatal intensive care unit (NICU)

	Total (*n*=46)	CRKP infection (*n*=21)	CRKP colonization (*n*=25)	*P* value
Sex, female, no.(%)	24(52.17)	11(52.38)	13(28.26)	0.97
Age, days, median(IQR)	2.00(13.21)	5.00(21.90)	1.00(6.46)	0.372
Gestational age, weeks, mean±SD	33.54±3.98	32.86±4.39	34.12±3.59	0.265
Body weight, kg, median(IQR)	2.135(1.63)	1.51(1.72)	2.23(1.63)	0.12
Height, m, mean±SD	0.43±0.07	0.41±0.08	0.44±0.05	0.11
Body mass index, kg/m^2^, mean±SD	11.21±2.62	10.79±2.72	11.55±2.53	0.42
Underlying condition, no.(%)				
Newborn respiratory distress syndrome (NRDS)	15(32.61)	7(15.22)	8(17.39)	0.92
Respiratory failure	29(63.04)	15(32.61)	14(30.43)	0.28
Lung infection	44(95.65)	20(43.48)	24(52.17)	0.90
Sepsis	14(30.43)	13(28.26)	1(2.17)	<0.001
Shock	9(19.57)	4(8.7)	5(10.87)	0.93
Pneumothorax	3(6.52)	2(4.35)	1(2.17)	0.45
Atelectasis	1(2.17)	1(2.17)	0(0)	0.27
Antimicrobial treatment, no.(%)				
Ticarcillin/clavulanate potassium	44(95.65)	19(41.30)	25(54.35)	0.11
Cefoperazone/sulbactam	8(17.39)	6(13.04)	2(4.35)	0.06
Ceftriaxone sodium	7(15.22)	5(10.87)	2(4.35)	0.14
Meropenem	7(23.91)	6(13.04)	1(2.17)	0.02
Imipenem	11(19.57)	10(21.74)	1(2.17)	<0.001
Vancomycin	9(19.57)	7(15.22)	2(4.35)	0.03
Amikacin sulfate	10(21.74)	7(17.39)	2(4.35)	0.03
Aztreonam	1(2.17)	1(2.17)	0(0)	0.27
Types of antibiotics used≥3	14(30.43)	12(26.09)	2(4.35)	<0.001
Hospitalization, days, median(IQR)	27.00(31.00)	40.00(38.5)	22.00(26.5)	0.06
NICU hospitalization, days, median(IQR)	23.50(24.25)	30.00(34.00)	22(17.5)	0.02
Hospitalization before CRKP isolation, days, median(IQR)	13.50(12.25)	15.00(15.00)	12(11.5)	0.40
Source of positive culture, no.(%)				
Sputum	29(63.04)	12(26.09)	17(36.96)	0.447
Catheter	6(13.04)	1(2.17)	5(2.17)	0.126
Secretion	1(2.17)	0(0)	1(2.17)	0.354
Shunt fluid	1(2.17)	1(2.17)	0(0)	0.270
Blood	6(13.04)	5(10.87)	1(2.17)	0.047
Urine	3(6.52)	2(4.35)	1(2.17)	0.450
Outcome, no.(%)				
Recovered(no.[%])	38(80.85)	16(34.78)	23(50.00)	0.010
Treatment abandonment	7(19.15)	5(10.87)	2(4.35)	0.137

### Antimicrobial resistance patterns among *K. pneumoniae* isolates

To investigate the antimicrobial susceptibility profile of clinical CRKP strains collected from NICU wards, we performed antimicrobial susceptibility testing and found that all CRKP isolates were resistant to carbapenem, cephalosporins, and penicillins. The resistance rates to aztreonam, ciprofloxacin, trimethoprim/sulfamethoxazole, nitrofurantoin, and doxycycline were 60.0%, 2.0%, 92.0%, 36.0%, and 8.3% respectively. These results therefore illustrated the emergence of multidrug resistance among CRKP isolates. Notably, all 46 strains showed sensitivity to amikacin, gentamicin, and colistin (Table 2).

**Table 2:** Antimicrobial susceptibility of isolated *K. pneumoniae* to antimicrobial agents

Antibiotics	Resistance, no. (%)	Intermediate, no. (%)	Susceptible, no. (%)	MIC50	MIC90	MIC range
Piperacillin	100.0	0.0	0.0	128	128	128–128
Cefoperazone/sulbactam	100.0	0.0	0.0	64	64	64–64
ampicillin/sulbactam	100.0	0.0	0.0	32	32	32–32
Ticarcillin/clavulanic acid	100.0	0.0	0.0	128	128	128–128
Piperacillin/tazobactam	100.0	0.0	0.0	128	128	128–128
Cefazolin	100.0	0.0	0.0	64	64	64–64
Cefuroxime	100.0	0.0	0.0	64	64	64–64
Ceftazidime	100.0	0.0	0.0	64	64	64–64
Ceftriaxone	100.0	0.0	0.0	64	64	64–64
Cefepime	100.0	0.0	0.0	64	64	32–64
Cefotetan	100.0	0.0	0.0	64	64	64–64
Cefuroxime	100.0	0.0	0.0	64	64	64–64
Aztreonam	60.0	0.0	40.0	64	64	1–64
Imipenem	100.0	0.0	0.0	32	64	16–64
Meropenem	100.0	0.0	0.0	16	32	16–32
Amikacin	0.0	0.0	100.0	2	2	2–2
Gentamicin	0.0	0.0	100.0	1	1	1–1
Tobramycin	0.0	0.0	100.0	1	1	1–1
Ciprofloxacin	2.0	0.0	98.0	0.25	0.25	0.25–0.25
Levofloxacin	0.0	2.0	98.0	0.25	0.25	0.12–0.25
Trimethoprim/sulfamethoxazole	92.0	0.0	8.0	384	384	1–320
Colisti	0.0	0.0	100.0	0.5	0.5	0.5–0.5
Nitrofurantoin	36.0	64.0	0.0	64	128	32–128
Doxycycline	8.3	0.0	91.7	4	4	2–16
Minocycline	0.0	2.0	98.0	2	4	2–4
Tigecyclin	0.0	4.0	96.0	0.5	1	0.5–1

### Antimicrobial resistance genes among CRKP isolates

**Fig. 1:**
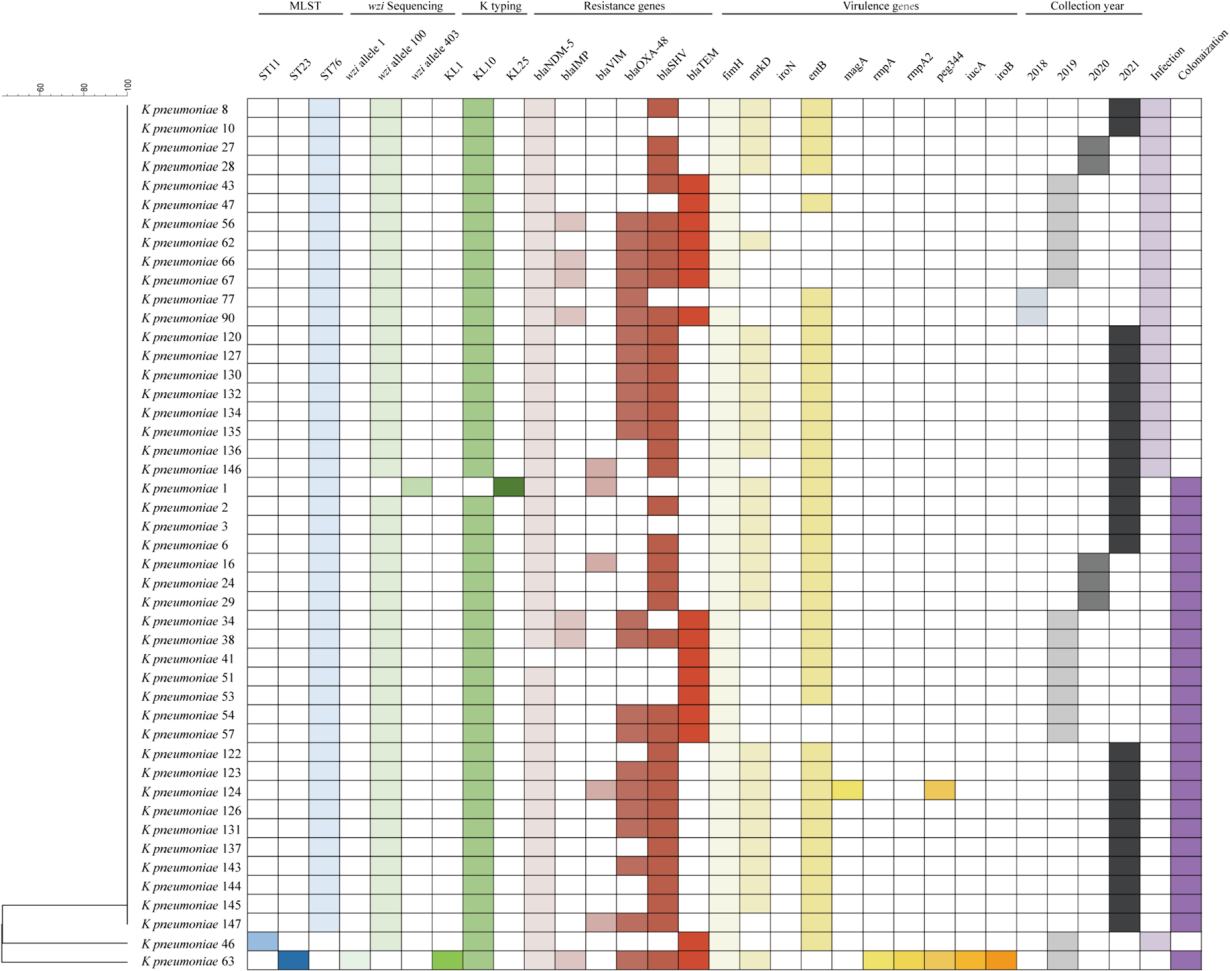
Epidemiology and molecular characteristics of the CRKP cases collected from NICU wards in Ningxia’s hospital between 2017 and 2021. Analysis of the phylogenetic relationship with BioNumerics software platform based on the distance matrix of pairwise differences between STs

Multiple β-lactamase genes associated with carbapenem resistance were detected in our study, including *bla*_NDM-5_, *bla*_SHV_, *bla*_OXA-48_, *bla*_TEM_, *bla*_IMP_, and *bla*_VIM_. Our results revealed that all CRKP isolates in this study expressed resistance genes (Fig. 1). The *bla*_NDM-5_ gene was detected in all 46 isolates (100.0%), while *bla*_SHV_, *bla*_OXA-48_, *bla*_TEM_, *bla*_IMP_, and *bla*_VIM_ genes were expressed by CRKP isolates at ratios of 78.26% (36/46), 60.87% (28/46), 34.78% (16/46), 15.22% (7/46), and 10.87% (5/46), respectively. Among the CRKP isolates, 97.83% (45/46) possessed at least two resistance genes, and 13.04% (6/46) CRKP strains co-expressed *bla*_NDM-5_, *bla*_IMP_, *bla*_OXA-48_, *bla*_SHV_, and *bla*_TEM_ genes.

### Virulence-associated genes among CRKP isolates

To evaluate the virulence potential of these clinical isolates, we conducted PCR tests to determine the presence of virulence-associated genes. The results are presented in Fig. 1. The majority of CRKP isolates tested positive for *fimH* (97.83%), *mrkD* (63.04%), and *entB* (82.61%). The detection rate for *magA*, *rmpA2*, *peg344*, *iucA*, and *iroB* genes was 2.17%. None of the isolates possessed *iroN* genes. Among the isolates, only *K. pneumoniae* 63 had all five virulence genes (*rmpA*, *rmpA2*, *peg344*, *iucA*, and *iroB*), while *K. pneumoniae* 124 also had the *peg344* gene.

### Wzi sequencing and K typing

Multiplex PCR analysis of 46 strains revealed that none of the isolates tested positive for K1, K2, K5, K20, K54, or K57, indicating that this method was unable to determine their K type. However, *wzi* sequencing confirmed that all isolates had identifiable wzi alleles associated with specific K types. Specifically, 97.83% (45/46) of the isolates were identified as *wzi*100-K10, while only one isolate was determined to be *wzi*403-K25.

### Multi-locus sequence typing and phylogenetic analysis

**Fig. 2:**
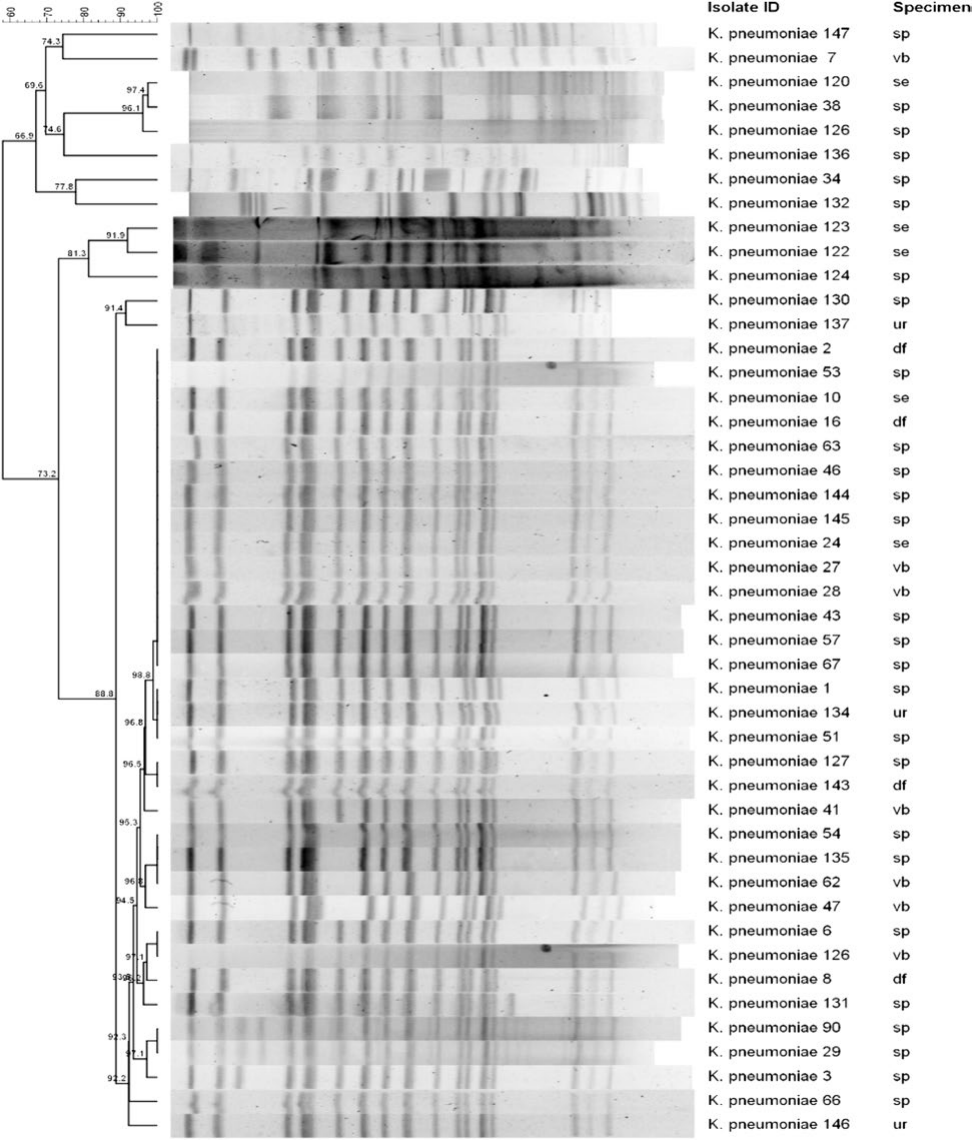
Pulsed-field gel electrophoresis profiles of 46 CRKP strains. The genetic similarity index scale is shown on the left side of the chromatogram. Isolate ID and specimen types are provided for each lane. Abbreviations: sp, sputum; ur, urine; df, drainage fluid; se, secretions; vb, venous blood

The molecular profiles of all strains were determined by pulsed-field gel electrophoresis (PFGE), and the sequence profiles of the strains were obtained through multipoint sequence typing (MLST) analysis. The dendrogram in Fig. 2 was generated based on PFGE analysis without weighting the STs included in the graph. All the isolates were classified into three clusters: A (isolate NO: 122, 123, 124), B (isolate NO: 130, 137), and C (2, 53, 10, 16, 63, 46, 144, 145, 24, 27, 28, 43, 57, 67, 1, 134, 51, 127, 143, 41, 54, 135, 62, 47, 6, 126, 8, 131, 90, 29, 3, 66, 146), based on a cutoff of 80% genetic similarity (Fig. 2). Unfortunately, 8 out of the 46 strains showed lower similarity compared to the others. Among the 46 CRKP isolates, three ST types (ST76, ST11, ST23) were identified (Fig. 1). ST76 was the most prevalent type, accounting for 97.83% of the isolates, followed by one ST11 (2.18%) and one ST23 (2.18%) isolate. The phylogenetic tree was constructed based on the combined gene sequences of MLST profiles, revealing two clusters. All ST76 isolates showed the same MLST profiles and belonged to the same cluster.

### Virulence detection in the mouse infection model

**Fig. 3:**
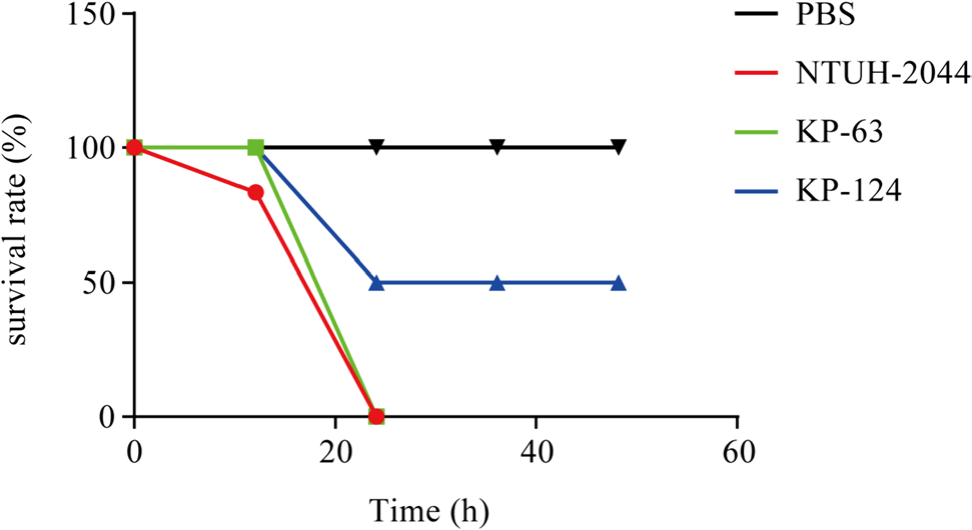
Virulence potential of clinical CRKP strains in a mouse infection model. The survival rate of mice was assessed after inoculating them with 5×10^7^ colony-forming units of each strain. The strains tested included two CRKP strains (KP-63, KP-124), one hypervirulent strain (NTUH-2044) as a positive control, and PBS as a negative control

In our study, we classified *K. pneumoniae* isolates carrying at least two of the six virulence genes (*iucA*, *rmpA*, *rmpA2*, *iroB*, *magA*, and *peg344*) as hvKP strains, namely *K. pneumoniae* 63 (KP-63) and *K. pneumoniae* 124 (KP-124). To further assess the virulence potential of KP-63 and KP-124 strains in vivo, we employed a mouse intraperitoneal challenge model. Our results demonstrated that the survival rate at 24 h was 0% for the K1 hypervirulent *K. pneumoniae* strain NTUH-2044 and KP-63, indicating comparable or higher virulence compared to the positive control NTUH-2044. Additionally, the survival rate at 24 h was 50% for KP-124 (Fig. 3).

### Whole-genome sequencing of KP-63 strain

**Fig. 4:**
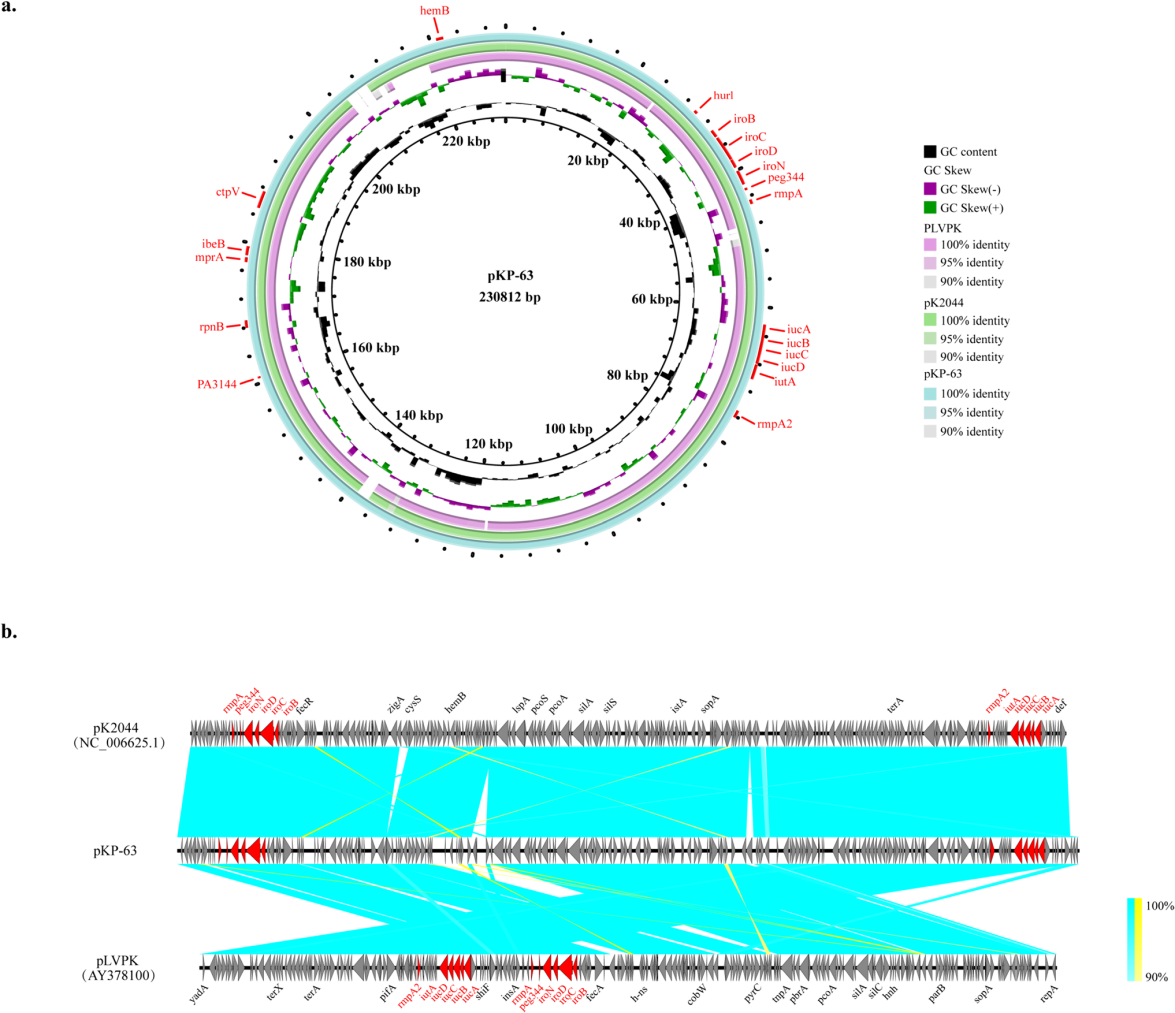
Comparative genomic analysis of the virulence plasmid from KP-63 strain. **a** Alignment of the approximately 230-kbp virulence plasmid recovered from hypervirulent carbapenem-resistant *K. pneumoniae* 63 strain (pKP-63) against two known virulence plasmids, such as pLVPK (Acc.no.: AY378100.1) and pK2044 (Acc.no.: NC_006625.1). The circular map was generated with the BLAST Ring Image Generator. **b** Linear genome alignment analysis among pKP-63, pLVPK, and pK2044 were generated based on EasyFigure software. Colored arrows indicate ORFs and the shaded region reflects sequence similarity. The virulence-associated genes are indicated in red

The ST23 CRKP strain, KP-63, showed a hypervirulent phenotype in the string test and mouse infection model, likely due to its possession of various virulence genes. To further investigate the characterization of the virulence plasmid in the KP-63, whole-genome sequencing (WGS) was performed using DNB system and Pacbio platforms. The results revealed a fully assembled plasmid in KP-63 (pKP-63), approximately 230 kbp in length, which displayed a strong similarity to the virulence plasmids pLVPK and pK2044 based on the Basic Local Alignment Search Tool (BLAST) analysis (https://blast.ncbi.nlm.nih.gov/Blast.cgi). Sequence alignment indicated a 99.41% identity and 93% coverage with pLVPK, and a 100% identity and 97% coverage with pK2044, suggesting a high similarity to pK2044 (Fig. 4a). Within the IncHI1B/repB virulence plasmid pKP-63, a region spanning from 24-kbp to 80-kbp contained multiple virulence-associated genes such as rmpA and rmpA2, responsible for mucoid regulation, and iroB, iroC, iroD, and iroN, which are involved in salmochelin production (Fig. 4a, b). These findings from WGS analysis were consistent with the results of the PCR assay for detecting virulence genes. Furthermore, the presence of conjugative transfer elements (https://tool2-mml.sjtu.edu.cn/VRprofile/) in pKP-63, such as an origin of transfer (oriT) region, type IV coupling protein (T4CP) genes, and transposase-encoding elements, suggested a potential horizontal transmission of virulence genes between different plasmids or isolates. In summary, these genomic results provide evidence supporting the identification of the hypervirulent ST23 CRKP strain in the NICU ward of a hospital in Ningxia province.

### Worldwide prevalence of sequence type 23 KP

**Fig. 5:**
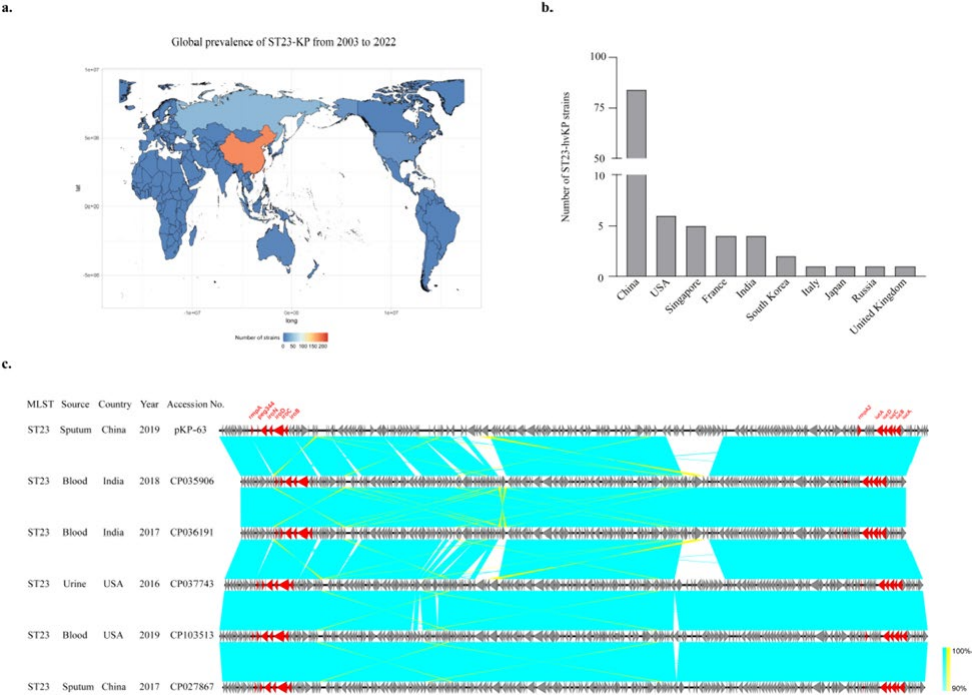
Global prevalence of sequence type 23 (ST23) KP and hypervirulent ST23-KP strains from 2003 to 2022. **a** Worldwide distribution of ST23-KP strains was mapped using the Hiplot research data visualization platform (https://hiplot.com.cn). **b** Analysis of statistics of ST23 hvKP strains in different regions. **c** Synteny analysis of hypervirulent *K. pneumoniae* virulence plasmids in ST23 strains reportedly from other global regions. Colored arrows indicate ORFs and the shaded region reflects sequence similarity. The virulence associated genes are indicated in red. The data collected from 2003 to 2022 were obtained from PATRIC database (http://www.patricbrc.org)

Based on the analysis of the MLST Database, the KP-63 strain has been identified as sequence type 23 (ST23) and capsular type K1, which is classified as one of the hypervirulent KP strains (hvKP) in clinical practice (Nakamura et al. 2021). The global distribution of ST23-KP strains showed that China had a higher number of strains compared to other countries and regions (Fig. 5a). Among the ST23-KP strains, the number of hypervirulent ST23-KP (ST23-hvKP) strains in different regions were as follows: China, 84; the USA, 6; Singapore, 5; France, 4; India, 4; South Korea, 2 (Fig. 5b). Furthermore, phylogenetic tree analysis revealed that ST23-hvKP strains from various global regions shared genomic similarity with plasmid KP-63 (Fig. 5c), implying dissemination of ST23-hvKP strain globally.

## Discussion

Severe bacterial infection is a significant cause of neonatal mortality, especially after antibiotic treatment, as it leads to the development and spread of multidrug-resistant bacteria. Moreover, the emergence of CR-hvKP poses a serious threat to the lives of newborns, presenting considerable challenges in clinical treatment and hospital infection prevention and control. In this study, we investigated 46 strains of CRKP isolated from neonatal wards in Ningxia’s hospital between 2017 and 2021, including one CR-hvKP strain. We analyzed their clinical and molecular characteristics to gain a better understanding.

In the present study, we isolated a total of 46 CRKP strains from 2017 to 2021. The number of strains isolated each year were as follows: 0 in 2017, 2 in 2018, 15 in 2019, 5 in 2020, and 24 in 2021 (Fig. 1). This data indicates an increasing trend of this strain, which may cause severe infectious diseases in newborns. Out of the 46 patients, 21 patients (45.65%) developed CRKP infection, leading to prolonged hospitalization in the NICU with a median duration of 15 days (IQR, 15.00). On the other hand, 25 patients (39%) acquired CRKP colonization without developing infection. None of the newborns in our study acquired subsequent nosocomial CRKP infection during the study period, but the colonization of CRKP may increase the incidence of CRKP infection (Qin et al. 2020). Multiple risk factors, including patient characteristics, environmental factors, previous microbiology status, and antibiotic exposures, have been suggested to be associated with increased CRKP colonization and/or infections (J. Wang et al. 2017). These risk factors are not only applicable to primary multidrug-resistant bacteria colonization, but also to the possibility of colonization and infection by other bacteria, which may require additional antibiotic treatment (Chang et al. 2021; Dai et al. 2021). Our study revealed that 76.20% (16/21) of newborns with CRKP infection had been treated with meropenem or imipenem prior to CRKP isolation, suggesting a correlation between antibiotic exposure and the emergence of resistant bacteria. Interestingly, we also observed that some patients treated with non-carbapenem antibiotics, such as ticarcillin commonly used for all colonized patients, developed CRKP colonization. This finding raises the possibility of horizontal transmission of CRKP through medical devices, close contact with healthcare workers, and other nosocomial sources. However, further verification is required, highlighting the importance of continuous monitoring of CRKP and the implementation of preventive measures in neonatal wards.

All 46 CRKP isolates showed high resistance to cephalosporins and carbapenems, of which 93.47% (43/46) belonged to the ST76-K10 clone carrying the *bla*_NDM-5_ gene. In China, the dissemination of CRKP strains has primarily been attributed to KPC-producing KPs, which mainly originate from the ST11 clone (Q. Wang et al. 2018; Zhang et al. 2020). However, our study found only one ST11-K10 strain co-producing *bla*_NDM-5_ and *bla*_TEM_ in the NICU. These findings are consistent with other studies that commonly observe the presence of the ST76 clone in neonatal wards. Nevertheless, the emergence of ST76 CRKP strains producing *bla*_NDM-1_ and *bla*_KPC-2_ has been recently reported in other provinces in China (Zhu et al. 2016). This indicates that the prevalence of CRKP can vary geographically, and there are genotype differences between newborns and adults.

Virulence potential was determined by detecting virulent-associated genes and employing mouse infection models. *Peg344* was found to be specific for hvKP, making it valuable for rapid diagnosis. In a study by T.A. Russo et al. (2018), it was confirmed that *peg344*, *iroB*, *iucA*, r*mpA*, and *rmpA2* accurately identified a strain as hvKp with 95% accuracy (Russo et al. 2018). In our study, we identified two *peg344*-positive strains: KP-63 and KP-124. Additionally, KP-63 co-harbored *iroB*, *iucA*, and *rmpA2*, and exhibited a comparable virulence potential to the hypervirulent NTUH-2044 in mouse infection models. WGS analysis demonstrated that pKP-63 exhibited a high genomic similarity to pK2044 and contained mobile elements capable of horizontally transferring these virulence genes, which is consistent with previous experimental findings. Similar to NTUH-2044, KP-63 belonged to the ST23 K1 serotype hvKP strain, which is known to be associated with severe pneumonia and liver abscess (Bei Li et al. 2014; Chung et al. 2008; Shon et al. 2013; Jane F. Turton et al. 2007). The global distribution of ST23-KP and ST23-hvKP strains is primarily observed in China, with the USA following behind. A comparative analysis of the virulence plasmids of ST23 strains from different geographic regions revealed a significant level of homology among them. The presence of key virulence genes, such as *rmpA*, *peg344*, and *iutA-iucABCD*, in all strains indicates their shared evolutionary ancestry.

As a CR-hvKP, the KP-63 strain is not only highly resistant to many antibiotics but also hypervirulent, which poses significant challenges to clinical treatment. However, despite being colonized with the KP-63 strain, the patient did not develop severe infectious diseases during hospitalization and eventually recovered after treatment. This can be attributed to the enhanced immune activation due to antibiotic treatment and good clinical care, which might have suppressed the growth of the colonized KP-63 strain. It is important to note that the KP-63 strain still has the potential to cause severe diseases when the patient’s immunity is compromised. Other studies have also reported similar findings of hvKP colonization (Lin et al. 2015; Yang et al. 2022). Nevertheless, it is crucial to implement routine surveillance and strict hygiene measures in the hospital to prevent the spread of clinical non-infectious isolates.

However, there are certain limitations to consider in this study. Firstly, the research was conducted using a limited number of cases and did not include any recent cases from 2022 and 2023. Secondly, the connection between the clinical outcomes of infections caused by predominant ST76 strains, with or without the presence of virulence plasmids, is still unclear. Lastly, it is essential to assess the risk factors for CR-hvKP infections in all hospital wards to predict epidemic trends and implement timely prevention and measures.

In conclusion, the prevalence of ST76 CRKP strains in the NICU ward in Ningxia’s hospital was confirmed with molecular epidemiology analysis. Also, we reported one hypervirulent ST23 CRKP strain identified by virulence phenotype assay and genomics analysis, which present challenges for treatment of infectious diseases caused by CR-hvKP strain.

## Data Availability

The data and material generated during and/or analyzed during the current study are available from the corresponding author on reasonable request.
